# The Female Global Scholars Program: A mixed-methods evaluation of a novel intervention to promote the retention and advancement of women in global health research

**DOI:** 10.1371/journal.pgph.0002974

**Published:** 2024-05-28

**Authors:** Alexandra A. Cordeiro, Kathleen F. Walsh, Radhika Sundararajan, Lindsey K. Reif, Margaret McNairy, Jyoti Mathad, Jennifer A. Downs, Sasha A. Fahme

**Affiliations:** 1 Center for Global Health, Weill Cornell Medicine, New York, New York, United States of America; 2 Department of Medicine, Weill Cornell Medicine, New York, New York, United States of America; 3 Department of Emergency Medicine, Weill Cornell Medicine, New York, New York, United States of America; 4 Department of Medicine, Weill Bugando School of Medicine, Mwanza, Tanzania; 5 Faculty of Health Sciences, Epidemiology and Population Health Department, American University of Beirut, Beirut, Lebanon; University of Washington Department of Global Health, UNITED STATES

## Abstract

Fewer than 25% of global health leadership positions worldwide are held by women, adversely impacting women’s health and widening gendered health disparities. The Female Global Scholars (FGS) Program, established in 2018 at Weill Cornell Medicine, is a two-year hybrid training and peer-mentorship program that promotes the retention and advancement of early-career female investigators conducting health research in low- and middle-income countries (LMICs). The purpose of this study is to determine the impact of the FGS Program on individual career advancement, academic productivity, and research self-efficacy. This mixed-methods study followed an explanatory sequential design. Participants completed an electronic survey collecting information on demographics, academic milestones, and research skill competency. Survey data were descriptively analyzed using R (Version 1.4.1106). In-depth interviews explored perceptions of the impact of the FGS Program on career development. The authors independently reviewed and thematically analyzed de-identified transcripts using NVivo (Version 13). In June 2022, twelve participants completed the survey. The median age was 40 years; 90% carried an MD, PhD, or other post-graduate degree. Since joining the FGS Program, respondents achieved a combined total of eight awarded grants, five academic promotions, 12 oral scientific presentations and 35 first-author peer-reviewed publications. Thematic analysis identified four overarching themes: gaining confidence through mimicry; improved self-efficacy to address gendered challenges; real-world application of scientific and career development skills; and building multi-disciplinary communities in a protected female-only space. We demonstrate that this low-cost training and mentorship program successfully addresses critical barriers that impede women’s advancement in global health research. Our data may inform the adaptation of this initiative across other academic institutions.

## Introduction

Women constitute the majority of the global healthcare workforce yet remain under-represented within global health decision-making roles [1]. Though 75% of trainees interested in global health are women, women hold only 25% of health leadership positions [2,3]. The exclusion of women from global health leadership is particularly evident among women from low- and middle-income countries (LMICs), who occupy just 9% of board seats across 146 global health organizations [4]. Women conducting global health research are uniquely positioned to provide critical insight and expertise on decisions related to women’s health programming, resource allocation and the delivery of services [4]. The under-representation of women in global health leadership therefore constitutes a critical gap with significant implications for women’s health outcomes.

Our prior work demonstrates that women confront unique challenges to reaching and maintaining health leadership positions, in part due to engrained gendered hierarchies, socio-cultural contexts around caregiving responsibilities, sexual harassment, and a lack of training opportunities [5,6]. Such challenges are most acute among junior female trainees, whose attrition from the field may be further compounded by the relative paucity of senior female mentors in global health [6]. Increasing the presence of women mentors could be invaluable to promoting the career advancement of junior female investigators and ultimately improving women’s representation in global health leadership. Female global health researchers are well-suited for health leadership positions as they not only possess knowledge of health disparities in diverse contexts, but also have distinct research and service priorities, which may profoundly impact health systems, policies, and planning [7,8].

The Weill Cornell Female Global Scholars (FGS) Program, a two-year leadership training and peer mentorship program, responds to these gaps and aims to promote the retention and advancement of early-career female investigators conducting global health research in LMICs. Informed by our formative qualitative research among female global health trainees in LMICs, the innovative hybrid curriculum focuses on mentorship, academic leadership, and scientific research skills. The FGS Program capitalizes on a network of senior female global health scientists to provide virtual and in-person didactic and interactive training, as well as opportunities for scientific presentations, collaborative grant-writing, and facilitated peer mentorship.

We have previously described the FGS Program objectives and curriculum [9]. The first year of the FGS Program includes intensive workshops to strengthen fundamental research skills and a longitudinal series on intersectional approaches to conflict resolution and academic leadership tailored to female investigators living and working in LMICs. The second year of the FGS Program centers around facilitated peer mentorship and networking at the participants’ respective home institutions to build capacity and develop practical leadership experience. Between April 2018 and September 2022, twenty-two early-career female investigators participated in the FGS Program. Bi-monthly virtual sessions continued throughout the COVID-19 pandemic.

The purpose of this mixed-methods study is to determine the impact of the Weill Cornell FGS Program on early-career female investigators’ career progression, academic productivity, and research self-efficacy. We offer recommendations for adapting this Program to other academic institutions and organizations in resource-limited settings to promote the advancement of women’s global health leadership.

## Materials and methods

### Study design and population

This study follows an explanatory sequential mixed-methods design. An anonymous electronic survey was sent by email to all former and current FGS Program participants (N = 22) in September 2021 and were completed between September 23^rd^, 2021 and October 12^th^, 2021. Twelve participants completed the survey, reflecting a 55% participation rate. Current participants (N = 13) were invited by email sent by an unaffiliated interviewer to participate in an in-depth interview. All current FGS Program participants agreed to take part in the interview.

### Survey and quantitative analysis

The electronic survey collected information on basic demographics, academic rank, professional achievements, promotions, feedback on individual webinars, and research skill competency using the validated Clinical Research Appraisal Inventory (CRAI) Scale, which was originally developed to assess research self-efficacy among early-career physician-scientist minorities, including women and people of color [10]. Using a ten-point Likert scale, the CRAI Scale measures self-rated confidence in research competency (0 = no confidence and 10 = total confidence) across the following research domains: designing and collecting; reporting, interpreting and presenting; conceptualizing and collaborating; planning; funding; and protecting [11]. We calculated composite mean scores with standard deviations (SD) for each research domain. Survey data were collected and managed using the Research Electronic Data Capture (REDCap) electronic data capture tools hosted at Weill Cornell Medicine [12,13]. REDCap is a secure, web-based software platform designed to support data capture for research studies, providing 1) an intuitive interface for validated data capture; 2) audit trails for tracking data manipulation and export procedures; 3) automated export procedures for seamless data downloads to common statistical packages; and 4) procedures for data integration and interoperability with external sources. Descriptive statistics were performed using R Software for MacOS (Version 1.4.1106).

### In-depth interviews and qualitative analysis

Semi-structured in-depth interviews were conducted in May 2022. Interviews were conducted in English by a professional female interviewer with previous qualitative research experience who was unaffiliated with the study team, the FGS Program, or Weill Cornell Medicine. The interviewer underwent intensive training on qualitative research methods, including interviewing, by study team members prior to conducting the interviews. She had no relationship with study participants prior to conducting the interviews, and explained to each participant prior to obtaining informed consent that she was independently hired as an objective third party without any professional interests or biases related to the study.

Interviews were recorded via teleconferencing using Zoom (Version:5.15.7) and lasted approximately 45 minutes. All participants provided verbal informed consent prior to their participation. Verbal informed consent, rather than written consent, was sought primarily for convenience in this minimal-risk study, as interviews were conducted virtually with participants in different countries. There is also ongoing debate regarding the added value of written informed consent in qualitative research following uncoerced, verbal informed consent, with some researchers arguing that written consent serves researchers, rather than study participants [14].

Interviews followed a topic guide, which was developed to enrich understanding of the subjects included in the survey. Open-ended questions explored attitudes, opinions, and beliefs regarding perceived quality of mentorship, impact of the FGS Program on career trajectory, responses to gender bias in professional and personal settings, approaches to conflict resolution, and feedback on the curriculum. The FGS Program curriculum is detailed in S1 Table. Data were transcribed and de-identified by a professional service unaffiliated with the study team. Transcripts were not returned to participants for comment and/or correction as the quality of the recording was high and the responses understood to be clear. Three study team members (AAC, KW, and SAF) coded all transcripts. Interview recordings were destroyed after transcription to preserve participant confidentiality.

Transcripts were imported into NVivo Software Version 13 (QSR International, Doncaster, Australia). Data analysis followed Braun & Clarke [15] framework for thematic analysis, using a six-phase step-by-step guide: (1) independent reading and re-reading of transcripts to conceptualize initial themes; (2) generating an initial codebook and systematically coding data collated into each code; (3) searching for themes and collating relevant data into themes; (4) reviewing and clarifying themes by group process, with additional in vivo coding of data which includes re-reading of transcripts; (5) refining specifics of each theme and generating clear definitions; (6) choosing representative quotations for each theme. The initial coding tree included parent codes such as “challenges balancing professional and personal obligations” and “perceived impact of program on career development.” There were initially fifteen parent codes, which were ultimately merged and refined to create four overarching themes. Data saturation, defined as the absence of new or additional information obtained in successive interviews, was discussed among four authors, including those who conducted coding, and felt to have been achieved. All authors agreed upon the final set of themes and illustrative quotations.

### Ethical approval

Ethical approval was obtained from the Institutional Review Board at Weill Cornell Medicine (21–04023533), including permission to seek oral consent. All study activities conformed to the principles embodied in the Declaration of Helsinki.

## Results

**Fig 1:**
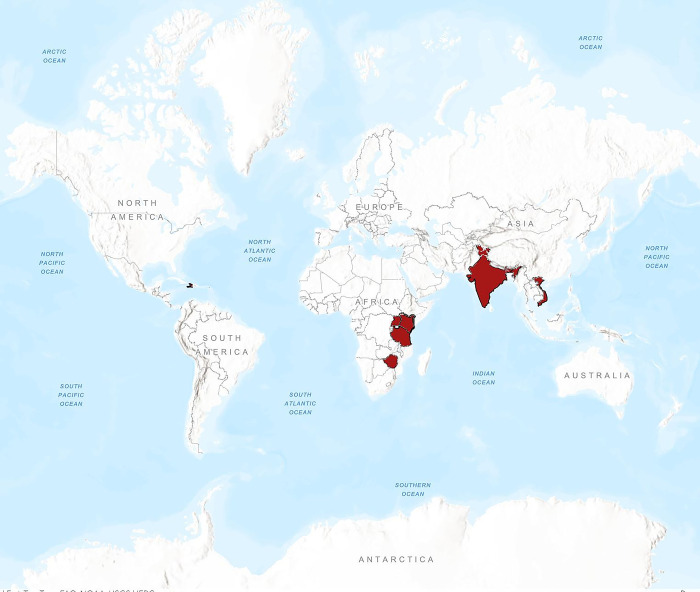
Countries of research among Female Global Scholars Program participants. At the end of 2021, thirteen participants were conducting global health research in seven different countries: Haiti, India, Kenya, Tanzania, Uganda, Vietnam, and Zimbabwe.

A total of 22 women participated in the FGS Program from 2018–2022. Seventy-two percent were from LMICs and 27% were from the U.S. **Table 1** reports basic demographic characteristics of participants who completed the self-administered survey (N = 12). Participants’ median age was 40 years. Ninety percent carried an MD, PhD, or other post-graduate degree. Participants conducted global health research in eight countries (**Fig 1**).

**Table 1:** Demographic characteristics of Female Global Scholars Program participants (N = 22) in 2022.

Participant Characteristics	N (%)
Median age (years) (IQR)	40 (32–44)
Currently married	20 (91)
Mean number of children (SD)	2.5 (1)
Highest degree obtained	
Bachelor’s	2 (9)
Master’s	11 (50)
MD and/or PhD	9 (41)

We identified four dominant themes and several sub-themes in our mixed-methods analysis. These findings are summarized in **Table 2**.

**Table 2:** Major themes and sub-themes from in-depth interviews with Female Global Scholar Program participants (N = 13) in 2022.

Themes	Sub-Themes
Confidence	Developing assertiveness and feeling optimistic about the future
	Gaining confidence by mirroring behavior of peers
Self-Efficacy	Empowerment to address workplace conflict
	Ability to balance work and family life
Research Career Advancement	Research skill competency
	Improved research productivity
	Real-world application of research and career development skills
Supportive Environment	Protected space for women
	Building multidisciplinary communities with other participants across countries

### Confidence

We noted an overall theme of confidence among participants. This includes participants’ perceived ability to advocate for themselves, feeling empowered to assume leadership positions, and having a positive outlook on their career advancement. Peer mimicry was identified an important mechanism of boosting confidence.

### Developing assertiveness and feeling optimistic about the future

Women expressed that the program allowed them to gain confidence in applying the leadership skills they learned to their careers in academic global health. Many explained that their participation in the FGS Program empowered them to advocate for themselves and take on greater responsibilities at their institutions:

*“[Through the FGS Program] …I learned how to be the real me*. *[Before the program] I didn’t take on a lot of responsibilities at work and I couldn’t express myself*. *Now*, *I do express myself [and believe] that it is up to me to show that women can do the same thing*. *This program…it doesn’t have a limit*. *I can do anything I want*.” - 7

*“Before [the FGS Program] I was in a cage*. *I would just sit back and be afraid to speak my mind*. *Now*, *through different webinars [and] mentorship…it’s made me come out to share*. *I’ve got to be able to speak up my mind*.*”*- 3

Women noted that the combination of didactic leadership training and practical experiences of developing and leading peer-mentorship networks at their respective institutions equipped them with the skills necessary to take on leadership roles. One woman detailed her experience, stating:

“*Joining the program…provided me [with] more confidence*, *and more reason…to move forward and try to achieve [my goals]* ….” She further explained how a meaningful mentorship relationship, complemented with the insights gained from other women in the field was especially critical for her:*“[The FGS Program] focused on women*, *and I think that was very beneficial for me… building that relationship*, *and also was empowering [me to be] a [role] model for other women who can also be leaders [in] what they are doing*.*”* - 9

Most participants conveyed a sense of optimism about their career in academic global health research after their participation in the FGS Program. They described how the FGS Program offered them a valuable opportunity to engage with senior female role models in the field. They reflected on how these interactions facilitated discussions about effectively balancing professional and personal challenges, while also fostering the development of their “soft skills” for networking:

*“The FGS Program helped me to empower myself*. *[The FGS Program] is like a peer support group…*. *I can tell you that gives me more ambition to move forward…sometimes you think you’re alone in this and now you realize there are a lot of us in the same boat…*. *this group was very helpful*.*”* - 4

### Gaining confidence by mirroring the behavior of peers

Many participants described how the FGS Program enabled them to effectively confront gendered challenges to maintaining academic productivity. They explained how the training and mentorship they received bolstered their confidence to remain committed to their research careers. One scholar expressed:

*“Women researchers have a lot to juggle*. *I really feel encouraged to see other moms*, *like me…pursue their dreams…that is truly an inspiration*. *Building my peer network has helped me see I can manage my plate too–I learn tactics they have used to balance competing priorities*. *I now have the confidence and motivation to make these tactics work for me too*.*”-* 13

One participant detailed how she appreciated that the FGS Program enabled her to meet and network with female leaders in the field. She explained how this experience was pivotal as these female role models shared similar motivations and suggested strategies for balancing a research career and family, which she found particularly encouraging:

“*The FGS Program also invited other women from [around] the world*, *very prominent people in…research…they talked about their experiences*, *they talked about their families*. *And it was very encouraging to see that the others have been there before me and*, *you know*, *I can also do it*.” - 6

Women described that it was helpful to mimic the behavior of other women, which later translated to actual application of leadership skills as they learned to nurture the growth and advancement of junior female investigators at their home institutions. One participant shared:

“*Before the program*, *I was a little bit shy to express myself when I’m in a meeting to express my thoughts…with my experience with the [FGS Program]*, *I find I am very different…now I have more responsibilities at work*. *And then*, *acting like a leader for other women in my institution*, *so I was always there to encourage them to express themselves*. *And then*, *to show that we women can be part of a scientific group*.*”* - 7

### Self-efficacy

Self-efficacy was found to be a major theme encompassing participants’ beliefs in their own capacity to successfully confront challenges in both their professional and personal spheres.

### Empowerment to address workplace conflict

Participants reported learning conflict resolution skills and feeling empowered to apply these skills effectively in the workplace. Several participants described successfully resolving interpersonal conflicts in the workplace:

*“We started from talking about…research goals…goals for life…family*, *you know*, *kind of getting to know each other a little bit more*. *And then we talked about… the specific issues that have been going on in the clinic*, *what are the issues*, *how would those issues make you feel*, *and do you think these are issues we can solve or not*, *and how*, *how do you think we can better support you to do your work*. *And it ended up being a really nice conversation…[I was able to] see her perspective of things and I was able to understand [her] issues…and I was also able to encourage her*. *And in that way…create a better environment for her*, *so I feel very successful*.” - 6

*“The conflict I’ve faced was really tough*, *[especially] when people don’t agree with each other…I didn’t want to interfere [in the past] because I didn’t want it to be as if I’m taking sides*. *It was really [hard] for me and it was actually affecting everyone else in the study because during meetings*, *if one [person] would say something then the other would just keep quiet and not say anything…I went back and reflected and thought about what we had talked about during the [FGS] discussions and I used what we had discussed to try to resolve the conflict*.*”* - 8

### Ability to balance work and family life

Women described that the FGS Program facilitated discussions regarding common challenges to balancing work and family life, including maintaining a productive research career while attending to family obligations. One woman noted:

*“I have two kids*. *And with that I have to also have a career and*, *you know*, *kind of manage home and manage work*. *And I think just being with other women who are also doing the same*, *and who are successful was super helpful*.*”* - 6

One participant explained how the FGS Program gave her adequate mentorship and skills to navigate work and tensions related to her extended family’s expectations. She described the FGS Program as a safe platform that allowed her to share her frustrations as well as seek advice and support from other women in similar positions. She cited how this contributed to her renewed sense of confidence to speak up about prioritizing her work:

“*The FGS Program [provides an] approach to managing the balance in the different areas*. *We talk about things like*: *‘What are my weaknesses*? *What are my strengths*?*’ … [The FGS Program] has helped me sometimes to… [have the] courage to explain [prioritizing my career] while not feeling guilty… the FGS Program has made me able to speak to my family*.” - 12

### Research career advancement

We identified research career advancement as a major theme. This included participants’ perceived competency in achieving fundamental research skills, improved research capacity, and the pragmatic application of research and career development skills.

### Research skill competency

**Table 3** displays the mean research skill competency scores captured by the CRAI Scale among survey respondents (N = 12). Participants reported relatively greater competency to conduct data collection, analysis, and presentation, than to acquire research funding.

**Table 3:** Research self-efficacy scores measured by the CRAI Scale among Female Global Scholars Program participants (N = 12) in 2022.

Clinical Research Domains	Mean Score (SD) out of 10 Maximum Points
Designing and collecting	7.2 (1.7)
Reporting, interpreting, and presenting	7.9 (1.5)
Conceptualizing and collaborating	6.5 (1.7)
Planning	6.8 (1.7)
Funding	4.5 (2.5)
Protecting	7.7 (1.9)
Overall CRAI Score	6.8 (2.2)

These survey data were supported by the in-depth interviews, in which several participants explained that participation in the FGS Program empowered them to continue and expand their research programs:

“*There are specific things…from the program that I think have been super helpful for me as [I have] advanced in my career…the talks that we’ve had*, *the people we’ve met…change how you view things and improve your confidence…the confidence to write grants*, *the confidence to write papers*, *the confidence to ask questions*.*”* - 6

*“[The FGS Program] taught me how to write manuscripts…and grants*. *In my country*, *we don’t know anything about those things but now I can try to write a grant and even if it’s not perfectly correct*, *I can try to write it*. *These are things women in my institution can do now*.*”* - 7

### Improved research productivity

**Fig 2:**
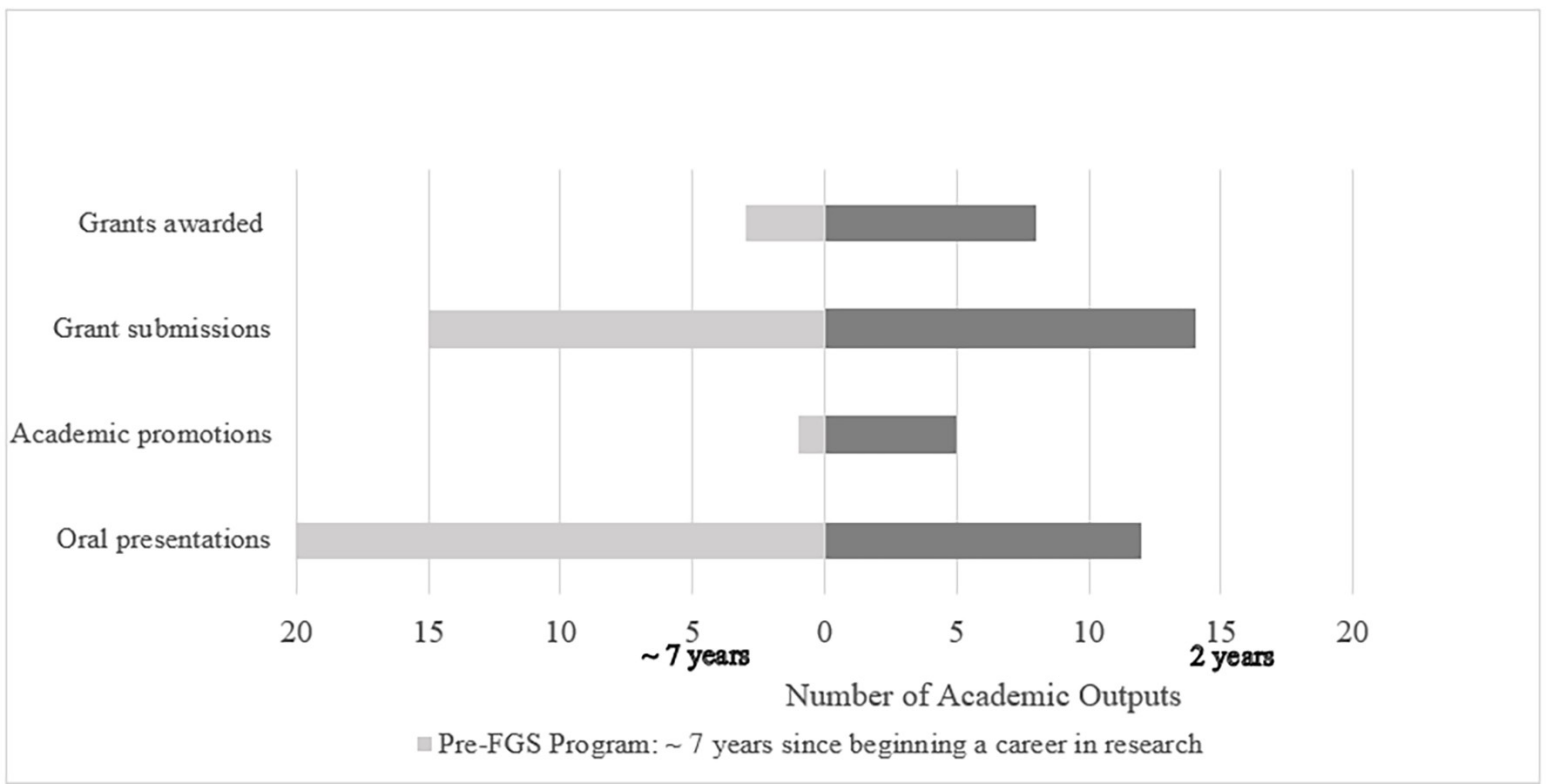
Participants’ research productivity pre- and post- Female Global Scholars Program.

Participants had been engaged in research for an average of seven years prior to joining the FGS Program. **Fig 2** highlights academic research outputs of 12 survey respondents comparing achievements prior to their participation in the FGS Program (~7 years) and after completion of the FGS Program (~1–2 years). Participants reported a cumulative 35 first-author peer-reviewed publications in high-impact journals including *AIDS*, *PLoS Global Public Health*, *JIAS* and *BMC Infectious Diseases* in the two years of program participation. Many scholars reported early-career milestones including poster presentations, mid-career achievements such as academic promotions, and grants awarded had increased since their participation in the FGS Program. Five scholars reported academic promotions, and eight were awarded extramural grants.

### Real world application of research and career development skills

Participants generally expressed an appreciation of the structure and curriculum of the FGS Program, emphasizing the importance of having gained valuable skills and knowledge beyond research skills:

*“The first year itself*, *it was about development of ourselves as women leaders…[through] sessions on manuscript writing and the sessions on how to make effective presentations*. *There was a session on how to write a proper biosketch…*.*I think that the way [the FGS Program was] structured was really helpful*.*”* - 11

One scholar described how the webinars enhanced her research skills, in particular sessions on how to effectively present research, to write abstracts, and to seek grants which were beneficial for the advancement of her career:

“*I had two opportunities of presenting my work*…*[through the FGS Program]*. *We got more skills [in] writing abstracts*, *writing papers*, *and then knowing how to effectively look for grants…I was at a stage of my research career [that] being part of this program…enabled me to acquire skills that empowered me*.*”* - 8

Scholars cited that the webinars were critical in providing a direct and tangible benefit for the advancement of their careers in their academic research. One participant explained how she leveraged the research skills she gained from webinars on writing a biosketch and defining the problem statement:

*“When writing a senior fellowship grant*, *the skills I learned in the FGS Program applied directly and helped me write effectively*. *When it came to articulating the problem statement*, *I referred to the session on specific aims*. *When it came to describing my expertise and my background as a candidate*, *I referred directly to the session on crafting a Biosketch*.*”* - 8

### Supportive environment

We found supportive environment to be a major theme, as participants repeatedly attributed many of their successes to having a protected space for women that uniquely facilitated collaborations and career advancement, particularly during challenging periods such as the COVID-19 pandemic.

### Protected space for women

Several participants found great value in that the FGS Program was an exclusive space for women, in which women were supported and affirmed as they navigated their careers. One participant appreciated that the FGS Program allowed her to connect and learn from both established female leaders in the field and fellow early-career investigators:

*“The fact that it was a space for women to see and share and learn together…the fact that [this] was a female [only] program*, *and the accessibility of the professionals and researchers and their diversity*, *made it really a place to be for me…*.*”* - 8

This participant further explained how the FGS Program served as a safe sanctuary, where she felt valued, recognized and celebrated, giving her a greater sense of empowerment. She commented:

*“*…*It was a really safe space for me… It was a safe place for women who want to grow*, *who want to open up…and be who you are*, *without fear of being judged*, *so it was a female space*.*”* - 8

Another participant shared how the FGS Program not only provided a protective and supportive environment for women to grow, learn and collaborate with one another:

“*I think it’s hard being a woman in most spaces in the world and I appreciated having a place to come and discuss things and try to problem solve*.*”* - 1

### Building multidisciplinary communities with other scholars across countries

Scholars cited that the FGS Program enhanced collaboration across organizational and cultural boundaries. One participant enthusiastically explained how she co-authored a publication with one of her fellow participants:

“*[Through the program] I have worked together with two other scholars in my district*. *We have worked together*, *published*, *and one [manuscript] is under review*.*”* - 3

Another participant reflected on the positive impact of international and female-centered collaboration for her career.

*“[I was] able to exchange ideas and views with fellow ladies in the program*. *I was able to get connections*.*”* - 8

The FGS community was described by many to be particularly helpful during the COVID-19 pandemic. Despite the professional setbacks described by many as a result of the COVID-19 pandemic, many women expressed that the FGS Program was beneficial in that it provided them a unique platform to escape their isolation and engage with other women who were facing the same challenges. They appreciated the sense of community the FGS Program brought despite them being in different countries. One participant shared:

*“Having other people from around the world…check in…in their various countries*, *how we are coping*, *was helpful*. *It was reassuring to see that each of us had…similar hurdles and challenges that we faced…I think bringing together a group from different places in the world was helpful just to see that we are not alone in the struggles*, *especially as a result of the pandemic*.*”* - 13

Another participant elaborated on how the FGS Program helped increase her research productivity during the pandemic:

*“Thanks to the FGS Program*, *I was able to keep connected and get ongoing mentorship*. *Even during the pandemic period*, *we had weekly webinars that kept us on track*, *that kept us engaged*.*”* - 8

## Discussion

We describe a novel leadership training and peer-mentorship program that successfully addresses known critical barriers that limit women’s career advancement in academic global health, including lack of female mentorship, limited training opportunities in global health research, and challenges of balancing work and family obligations [3,16]. Participants described enhanced academic research productivity since joining the FGS Program, and cited improved confidence to take on leadership positions, increased self-efficacy to address challenges related to work-life balance, and the practical application of scientific research skills gained as major benefits to participation. Our findings suggest that coupling peer mentorship with leadership and research training opportunities in female-dedicated spaces may mitigate some of the challenges which lead to the attrition of women from academic global health.

FGS Program participants noted improved academic research productivity following program enrollment. Supporting women’s research productivity is one of the main objectives of the FGS Program, as gender disparities in academic promotions, first-author publications, and awarded grants persist. Advancements towards gender parity are critically important given data that women and men leaders have different priorities [8], and that women physicians provide different, and in some cases superior, clinical care than men physicians [7,17–20]. Our findings also suggest high clinical research self-efficacy among FGS Program participants, as demonstrated by the self-rated research competency scores on the CRAI Scale. The CRAI Scale has been utilized in numerous studies to evaluate the effect of clinical research training programs on research self-efficacy among early-career physician-scientists [21,22]. While the mean CRAI Scale scores of FGS Program participants were comparable to–and in some cases higher than–those reported among early-career investigators at U.S. academic institutions [11,21], we notably demonstrate lower scores in research funding self-efficacy, potentially reflecting funding disparities which disproportionately impact LMIC-based global health researchers [23,24]. While participants of the FGS Program celebrated academic success prior to joining the program, these achievements such as conference presentations were predominately at the earlier stages of their careers. After joining the program, participants indicated an increase in their attainment of career milestones including academic promotions and successful grant submissions. Although causality cannot be determined in this study, qualitative data suggest that participation in the FGS Program contributed to these successes.

The FGS Program addresses a critical gap in mentorship for female trainees, which we have previously shown to be a significant predictor of attrition from the field [2]. Poor or absent mentorship frequently fosters dissatisfaction and discouragement for early-career female investigators, making it challenging to initiate a productive research career [25–27]. Recognizing that senior female scientists in academic global health may have limited protected time for mentorship, the FGS Program provides training designed both to optimize participants’ relationships as mentees with their primary scientific mentors, and to hone their own mentorship skills as they become peer-mentors to other program participants and begin to mentor junior investigators within their institutions. The transformative effects of mentorship for female trainees have been repeatedly demonstrated [26–30]. A longitudinal study conducted in Australia by Gardiner and colleagues [31] demonstrated that early-career women with mentorship support were more likely to be retained in academia, receive grants and have increased academic self-efficacy compared to non-mentored women [30]. Our findings suggest that similar benefits can be attained through a peer-mentorship program, which may be an effective, low-cost model to complement primary mentorship structures.

The novelty and success of the FGS Program is in part due to the establishment of a dedicated female space for global health researchers to build community with their peers. Participants cited this as a unique and helpful feature of the FGS Program, highlighting the sense of solidarity it created among participants and underscoring the importance of judgement-free spaces in academia, where women feel comfortable engaging with one another. Solidarity-building, which typically is not prioritized in training programs, has been shown to promote productive collaborations and is often a deliberate outcome of female-exclusive programs [32]. Leadership programs in other disciplines similarly and successfully adopted a women-only model, with participants citing greater freedom of expression and opportunities for self-reflection and growth in the absence of male oversight [33]. In our study, participants found it encouraging to learn about the journeys of women who face similar challenges and described mimicking their peers as a means of advancing their own research careers. A recent behavioral study among female college students demonstrated that exposure to female role models who exhibited confident body postures caused the students to emulate postures, in turn leading to increased student self-confidence, performance and empowerment [34]. Our findings indicate that mimicking respected peers may have a similar positive effect, and suggest that increasing the visibility of early-career female investigators may be a powerful means of empowering women in global health research.

The FGS Program additionally responds to ingrained socio-cultural norms which impede women’s career advancement in global health research [3,7,35,36]. It is well-established that women take on greater responsibilities at home compared to their male counterparts, and more often face career setbacks associated with motherhood and childrearing responsibilities [35,36]. Moreover, global health career paths frequently require women to spend extended periods of time abroad either to receive training not available in their home countries or to conduct research, making it difficult for them to fulfill family responsibilities [2]. Women from LMICs face even steeper obstacles to career advancement often rooted within patriarchal and white hegemonic cultural norms [37–39]. In these contexts, women often find themselves with limited power both to navigate and negotiate into positions of leadership [40]. Indeed, a recent systematized review called to attention not only gendered challenges, but also the racial, economic, and sociopolitical barriers which define global health systems and which disproportionately restrict the retention and advancement of LMIC-based women of color, advocating for an intersectional approach to achieving equity in global health leadership [40]. Through an interactive, intersectional leadership training series led by a young African woman and activist, the FGS Program aimed to address the nuanced challenges to leadership that uniquely impact women researchers living and working in the Global South. Many participants perceived that this series improved their self-efficacy to confront gendered conflict inside and outside of the home and navigate the complexities of race and gender in health leadership representation.

Importantly, while the FGS Program aims to address gendered barriers to global health research career advancement and representation at the individual- and peer-level, the intervention does not confront the institutional and sociocultural structures which prioritize male advancement into positions of health leadership. Such systemic barriers, which persist even in meritocratic fields, have been described by investigators across disciplines and geographic regions [33,41–43]. For instance, a qualitative study of early- and mid-career female academics in an Australian university showed that younger women’s career progression was deterred by the masculine culture defining academic leadership, which created a stressful and disempowering work environment for women [41]. Indeed, a major critique of women-specific programs is that there is an inherent assumption that women themselves are at fault, and that such initiatives offer a compensatory training that conforms with patriarchal institutional norms and values [41,42]. While the FGS Program does not directly confront institutional policies, which may dissuade women’s leadership participation, it seeks to equip women with a skillset essential to navigate such policies and to overcome ingrained disparities in order to achieve parity in leadership. **Table 4** outlines evidence-based, cost-effective recommendations based on our experiences implementing the FGS Program, to guide the development of similar initiatives in low-resource settings.

**Table 4:** Recommendations to guide the development of similar programs to support, retain, and advance early-career female scientists in low-resource settings.

Recommendation	Evidence-Based Benefits	Cost (Low/Medium/High)
Establish female-exclusive spaces for women trainees to come together	• Fosters a sense of community among women of diverse demographic and academic backgrounds within a safe environment, empowering women to speak freely [32,41].	Low
Support the development of local peer-mentorship networks	• Responds to feelings of isolation in academia and provides opportunities for peers to share insights on common challenges, such as balancing work-family life [44,45]. • Facilitates research collaborations and may promote academic productivity through joint publications, presentations, and grant submissions [46,47].	Low
Facilitate networking opportunities with senior female scientists from diverse demographic backgrounds	• Empowers trainees to pursue leadership positions by increasing the visibility and accessibility of successful role models [16,48,49].	Low-Medium
Adopt an intersectional approach to address not only gendered, but also racial, ethnic and socioeconomic barriers to women’s career progression	• Fosters inclusivity and validates women’s experiences, which may be shared across disciplines and regions [32,40].	Low

This study has several strengths. We utilized a mixed-methods approach to develop a more holistic understanding of the impact of the FGS Program on participants’ career development. In-depth interviews were an effective medium for exploring similarities and differences in participants’ attitudes and beliefs across diverse academic and demographic backgrounds. This study is subject to certain limitations. Firstly, the response rate for the survey was fairly low, which may have introduced selection bias to our quantitative findings. While we speculate that non-response was greater among FGS Program participants who had already completed the program, the anonymous nature of the surveys makes it difficult to ascertain differences in characteristics among respondents and non-respondents. Secondly, though the sample size of the qualitative component of this study was small, we were nonetheless able to achieve data saturation from in-depth interviews. Additionally, the results may be subject to social desirability bias. We attempted to mitigate this bias by anonymizing the electronic survey, employing an independent consultant to conduct the in-depth interviews, and emphasizing anonymity to participants. Notably, we did not comprehensively document the breadth of challenges experienced by participants, such as disparities in funding opportunities. Finally, while our findings are promising, prospective data are needed to examine the impact of the FGS Program on research self-efficacy and academic productivity, as the pre- and post-FGS Program participation time periods included in our analysis were varied. As a result, the achievements observed may be individualized and differ among participants.

## Conclusions

The overwhelming exclusion of women from global health decision-making not only jeopardizes the potential for improved care for women and children but also threatens to undermine progress towards Sustainable Development Goals (SGDs) 5 and 3, which aim to achieve gender equality and ensure the health and well-being of all communities [50]. Increasing the visibility and representation of women in global health leadership can have transformative effects on health outcomes for women, their families, and entire communities. We demonstrate the success of implementing a novel low-cost intervention to promote and retain women in global health research. The FGS Program can serve as a model for interdisciplinary research, leadership and career development programs for early-career women conducting health research in LMICs.

## Supporting information

S1 Checklist COREQ (Consolidated Criteria for Reporting Qualitative Research) checklist. (PDF)

S2 Checklist (DOCX)

S1 Table Overview of Female Global Scholars Program curriculum. (DOCX)
