# Supplementary material for: The Female Global Scholars Program: A mixed-methods evaluation of a novel intervention to promote the retention and advancement of women in global health research
Source: PLOS Glob Public Health. 2024 May 28;4(5):e0002974. doi: 10.1371/journal.pgph.0002974 (PMC11132512; doi:10.1371/journal.pgph.0002974)
Supplement: S1 Table — (DOCX) [file pgph.0002974.s003.docx]

| Topic Series | Description |
| --- | --- |
| Writing a biosketch and designing a concept sheet | Lectures on the essential skills required to develop and write a biosketch, which is crucial for NIH grant applications, and designing a concept sheet to concisely present research ideas tailored to individual research areas. |
| *Intersectional Leadership Series:*  1: Decolonizing Global Health | Overview with case-based discussions into the perspective of decolonizing global health. The series explores historical context of global health work and identifies approaches to conduct equitable and culturally sensitive research. |
| Building and nurturing mentor-mentee relationships | Understanding the dynamics of mentorship, the benefits and challenges of mentorship, and how to facilitate productive mentor-mentee relationships. |
| Introduction to Publishing | Overview to the publication process in academic research, authorship. Lecture covers basics of finding a target journal (including a mix of low, medium and high-impact journals) for scholars’ particular research area and skills to become a good peer reviewer. |
| Presentation Skills | A lecture series on how to give effective presentations and how to deliver concise and compelling elevator pitches. Scholars film their elevator pitches, which are reviewed and workshopped by their peers. |
| Manuscript-Writing | A lecture series on the art of manuscript writing, and how to develop skills for the production of effective and compelling academic research papers. |
| *Intersectional Leadership Series:*  2. Conflict Resolution | Overview of conflict resolution and how to develop valuable skills for effectively resolving conflicts in both professional and personal contexts. Workshops are designed to gain a deeper understanding of conflict dynamics and strategies to manage disputes and navigate challenging situations. |
| Building mentorship networks | Lecture series on strategies to build and maintain effective peer-mentorship networks and expand professional networking skills. |
| *Intersectional Leadership Series:*  3. Navigating Social Identities in Global Health Research | Interactive workshops exploring the intersection of social identities, including race, gender, ethnicity and the impact these factors have on women’s experiences in global health research. |
| Women in Global Health Research Initiative Scientific Conference | Attending and participating in the Weill Cornell Women in Global Health Research Scientific Conference, showcasing women’s research and expanding networks. |
